# Bilateral traumatic distal femoral transphyseal fracture in a 9-year-old male

**DOI:** 10.1093/jscr/rjaa572

**Published:** 2021-01-18

**Authors:** Timothy P Davis, Rujuta Mehta, Arpit Agrawal

**Affiliations:** Department of Trauma & Orthopaedics, King’s Mill Hospital, Sherwood Forest NHS Foundation Trust, Sutton-in-Ashfield, United Kingdom; Department of Paediatric Orthopaedics, Bai Jerbai Wadia Hospital for Children, Mumbai, Maharashtra, India; Department of Paediatric Orthopaedics, Bai Jerbai Wadia Hospital for Children, Mumbai, Maharashtra, India

## Abstract

A case of bilateral traumatic distal femoral Salter-Harris Type I fracture presented to our emergency department. History was of a 9-year-old male playing at a building site when a concrete block fell from height on to his knees, which were extended in a sitting position. Management was with analgesia and transfer to theatre followed by closed reduction and internal fixation—position was assessed under mobile X-ray. The patient made a full clinical recovery within 18 weeks and was followed-up over 5 years. There was no clinical effect on final adult length of femur and no deficit in range of movement. The foot-drop observed at presentation resolved over a period of 12 weeks. This case highlights the importance of performing a thorough neurovascular examination of the patient at presentation, followed by a careful closed reduction and internal fixation under anaesthesia, being careful not to damage the distal femoral growth plates.

## INTRODUCTION

Distal femoral physeal injuries account for 2% of all physeal injuries [1]. However, complications from these injuries requiring further surgery after initial management range in incidence from 40 to 60% [1]. Thus, knowledge of how to manage these fractures for long-term success is important. We present an unusual case of an ambulatory, otherwise-healthy, 9-year-old patient who presented within 6 h of a traumatic bilateral distal femoral Salter-Harris Type I fracture, resulting in epiphyseal slips. It covers the reduction manoeuvre and a good long-term follow-up. Similar cases reported in the literature are mainly unilateral [1, 2]. Two bilateral cases were reported: one in a bed-bound patient [3], and one in an ambulatory, healthy child, although follow-up was not reported [4]. We present a case of bilateral traumatic distal femoral transphyseal fracture in a 9-year-old male with a good outcome.

## CASE REPORT

**Figure 1:**
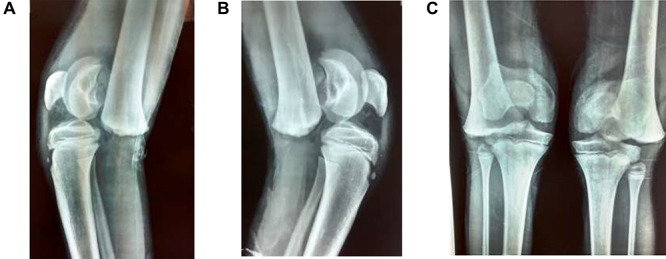
(**A**) Lateral view of right knee at presentation, (**B**) lateral view of left knee at presentation, (**C**) frontal view of both knees at presentation

A 9-year-old male presented to the ER within 6 h of injury to both knees, with swelling, restricted movements and tenderness bilaterally. Assessment demonstrated distal pulses in both legs. Initial management was with pain relief—neurological status was not assessed due to pain. A history of direct fall of a concrete block onto both knees whilst sitting with legs extended and raised on a stool at a building site, leading to a forced hyperextension injury, was elicited. The patient received no treatment before presenting to our unit. Radiography suggested bilateral completely displaced Salter-Harris Type I fractures (Fig 1A–C). All routine laboratory and metabolic profiles performed were within normal ranges. Patient was transferred to theatre immediately after radiography for a closed reduction.

**Figure 2:**
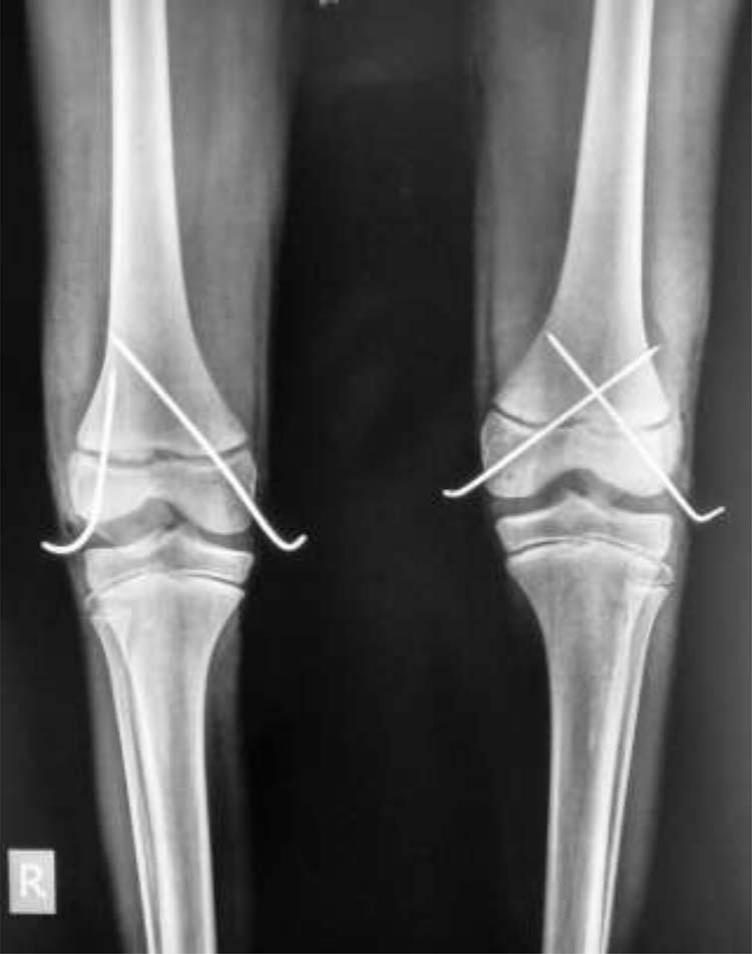
Frontal view of both knees after closed reduction and K-wire fixation

**Figure 3:**
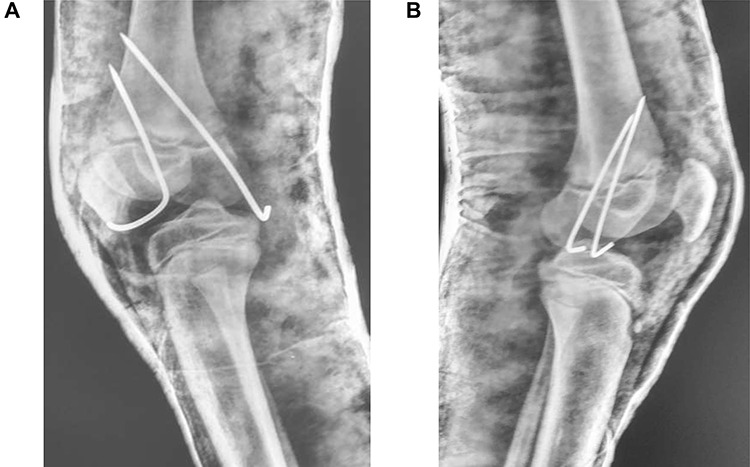
(**A**) Lateral view of right knee after closed reduction, K-wire fixation and anterior slab application; (**B**) Lateral view of left knee after closed reduction, K-wire fixation and anterior slab application

In theatre, general anaesthesia and prone positioning were performed. Closed reduction with gentle traction was achieved, followed by gradual flexion of the knee (Wilkin’s Manoeuvre). The physeal fragment was reduced with both thumbs, and reduction checked under mobile X-ray—good realignment was confirmed. Final fixation was achieved with percutaneous smooth K-wires in a cross construct (Figs 2, 3A and B). An anterior slab maintaining knee flexion was then applied. This process was repeated contralaterally. After 24 h and with adequate analgesia, bilateral foot-drop was noted. Bilateral posterior slabs were then applied.

Four weeks of regular analgesia and slab immobilization were followed by K-wire removal, splinting with bilateral ankle-foot orthosis (AFO) and physiotherapy, comprising of gradual range of motion exercises, and partial weight-bearing between 8 and 12 weeks. Full weight bearing was achieved by 12–16 weeks. Nerve conduction studies demonstrated common peroneal nerve re-innervation at 6 weeks, with full clinical recovery by 12–16 weeks’ post-injury.

**Figure 4:**
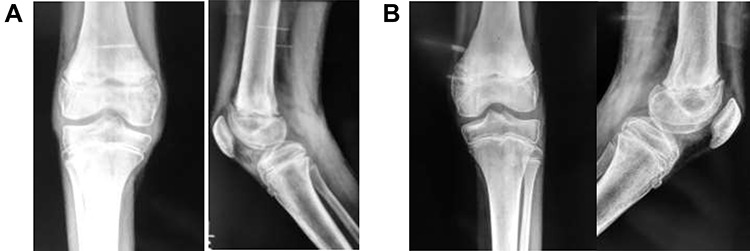
(**A**) Frontal and lateral view of right knee at 6-months post-injury; (**B**) frontal and lateral view of left knee at 6-months post-injury

Follow-up was every 6 months over the next 5 years (Fig. 4A and B). Full range of motion was achieved in both limbs, with the child fully ambulatory and with no leg-length discrepancy. Future adult femur length does not appear to have been impacted.

## DISCUSSION

The distal femoral physis is the largest and fastest growing physis in the human body—it contributes 35% of eventual leg length [5], and 70% of eventual femoral length [6]. Comparatively, the proximal femoral epiphysis contributes 15% to eventual leg length [6]. Distal femoral physeal injuries account for 2% of all physeal injuries [1], making this an uncommonly injured site. However, the incidence of complications requiring surgery after these injuries ranges from 40–60% [1]—knowing how to initially manage these fractures is essential for achieving long-term positive outcomes.

Salter-Harris Type I fractures of the distal femur are most common in neonates due to indirect trauma, through birth trauma or abuse, and adolescents due to direct trauma, through sporting-injuries or road-traffic collisions [1]; our patient does not fulfill either of these categories.

Unilateral slipped distal femoral epiphysis incidence is higher in our patient’s age set, due to metabolic (such as scurvy) [3] and generalized bone-weakening conditions (such as leukemia and myelodysplasia) [6]. Indeed, bilateral epiphyseal slips have been reported in the literature in patients with scurvy [3]—our patient’s Vitamin C and Calcium levels were normal.

Previously, in patients presenting acutely with distal femoral epiphyseal slip, closed reduction under general anaesthesia was deemed adequate management. If a fracture is completely stable on reduction, with no displacement, then immobilization alone suffices. However, immobilization alone is not the treatment of choice for high velocity injuries in which strong muscular forces are acting across a joint, increasing the likelihood of a late displacement [7]—displacement rates after our patient’s injury and treatment with immobilization alone are reported to be 43–60% [8]. Current best practice is closed reduction and percutaneous pinning. This algorithm was followed carefully bilaterally, to avoid fracture over-manipulation and further damage to the physes or neurovascular structures. Gentle manipulation and fixation was attempted successfully—no physeal damage has been determined since the operation took place. The initially-missed neurological symptoms highlight the risk of associated neurovascular damage in the popliteal fossa with this mode of injury. Fortunately, nerve-conduction studies showed common peroneal nerve reinnervation at 6 weeks with full clinical recovery at 12–16 weeks. This neurological deficit was likely due to a neuropraxia, which usually resolves over 6–12 weeks with appropriate splinting—as demonstrated by the nerve conduction studies. If the foot-drop had not returned to normal within 3 months, exploration of the nerve to check continuity would have been required, followed by nerve grafting if appropriate. The risk of neurological injury in conjunction with a distal femoral transphyseal slip is recognized in the literature—common peroneal nerve injury incidence is 2–7% [9, 10].

As it was a bilateral injury, gentle physiotherapy was started after plaster removal—only active and assisted-active movement was used, rather than force. Other studies have encouraged early physiotherapy post-plaster removal to reduce the risk of fixed-flexion deformity [3]—some suggest caution in using forcible manipulation for deformity correction [1]. The patient’s recovery has been excellent (Fig. 4A and B); long-term results demonstrate a full range of movement bilaterally with no distal neurological deficits. The patient can walk unassisted, has been observed running and is reportedly back to all routine activities.

## CONCLUSION

This case is rare in the number of limbs affected and follow-up length—few similar cases are in the literature. It shows that early careful closed reduction and fixation of these injuries is safe and effective management, after thorough clinical examination—supported by the long-term outcome. Of note is: the importance of adequate analgesia at initial presentation; the importance of thoroughly evaluating the patient’s distal neurovascular status; confirmation of the safe use of Wilkin’s manoeuvre.
